# “Words are too small”: exploring artmaking as a tool to facilitate dialogues with young South African women about their sexual and reproductive health experiences

**DOI:** 10.3389/frph.2023.1194158

**Published:** 2023-08-11

**Authors:** Felicity Hartley, Lucia Knight, Hilton Humphries, Jill Trappler, Katherine Gill, Linda-Gail Bekker, Virginia MacKenny, Jo-Ann S. Passmore

**Affiliations:** ^1^ Institute of Infectious Diseases and Molecular Medicine, University of Cape Town Medical School, Observatory, Cape Town, South Africa; ^2^ DSI-NRF CAPRISA Centre of Excellence in HIV Prevention, CAPRISA, University of Cape Town, Cape Town, South Africa; ^3^ Division of Social and Behavioural Sciences, School of Public Health & Family Medicine, University of Cape Town, Observatory, Cape Town, South Africa; ^4^ School of Public Health, University of the Western Cape, Bellville, South Africa; ^5^ Human Science Research Council, Sweetwaters, Pietermaritzburg, South Africa; ^6^ Department of Psychology, University of KwaZulu-Natal, Pietermaritzburg, South Africa; ^7^ Private Practitioner, Orange Art Project, Cape Town, South Africa; ^8^ Desmond Tutu Health Foundation, University of Cape Town, Cape Town, South Africa; ^9^ Michaelis School of Fine Art, University of Cape Town, Cape Town, South Africa; ^10^ National Health Laboratory Service, University of Cape Town, Cape Town, South Africa

**Keywords:** adolescent health, young women, sexual and reproductive health, artmaking, stigma, sex education

## Abstract

**Background:**

Adolescents and young women are at high risk for sexually transmitted infections (STIs) and unintended pregnancies. However, conversations about sexual and reproductive health (S&RH) are difficult and stigmatised. Visual art-based approaches have been a useful adjunct to language-dependent interviews, encouraging embodied memory recall. Here, we explored a novel visual art-based methodology—“Stories from the Edge”—with a cohort of young women to understand how artmaking might facilitate dialogue of how S&RH experiences influenced behaviour, to enrich dialogues captured in the individual in-depth interviews (IDIs).

**Methods:**

Seven isiXhosa-speaking young women (aged 21–25 years) were recruited into a six-session art-based engagement, painting the stories of their S&RH experiences. Large format artmaking and IDIs contributed to the data set. IDIs were audio recorded, transcribed, and translated and then analysed thematically.

**Results:**

Young women felt that the visual art-based methodology eased barriers to communicating experiences of S&RH-seeking behaviours, with one woman commenting that “words are too small” to capture lived experiences. Artmaking provided the opportunity to express emotional complexities of the pleasures of intimate relationships and the heartbreak of betrayal for which they had no language. Significant social relationships (family, partners, peers) influenced sexual and reproduction attitudes and practices more than healthcare facilities and staff and more distal socio-cultural attitudes/practices. These influences shifted from adolescence to adulthood—from family to peer and partners.

**Conclusion:**

Young women valued using the art-based methodology, which facilitated recall and verbalising their S&RH experiences more fully than language-only research. The process outlined here could provide a creative method that builds communication skills to negotiate the needs and desires of young women with partners and staff at S&RH services.

## Introduction

Adolescent girls and young women in Southern Africa are at high risk of both sexually transmitted infections (STIs) (1) and unintended pregnancies (2). Sexual reproductive health (S&RH) is difficult to talk about during adolescence, because of fear of judgement and shame in discussions with parents, caregivers, partners, or healthcare providers (3, 4). However, constructive conversations about S&RH are known to improve knowledge and S&RH outcomes (5), and creative approaches to engage, facilitate, and ease such conversations are urgently needed as these could promote the ability of young women to address their S&RH concerns. Language taboos and behaviour are shaped by cultural contexts, gender roles, and power that make communicating about S&RH challenging, even in adults (6). It is therefore likely that these same power dynamics within the lived experiences of young women in South Africa will impact the access of young women to S&RH resources and navigating these barriers could empower young women with the communication tools to promote their own S&RH.

Visual artmaking has been used in research in both humanities and sciences, including social, cultural, educational, and health for centuries (7, 8). The relationship between art and science throughout history has been complex and multifaceted, with the two fields having been closely intertwined at times, influencing and inspiring each other, while at other times being perceived as separate domains with distinct methodologies and goals (9). Here, we attempt to challenge the current status quo that the disciplines are separate and open a dialogue on the value that each discipline brings to human health and discourse. Visual art-based methods can support the development of communication skills to overcome some of the barriers young women experience when expressing their S&RH desires and needs (7, 10, 11) so that they can practise safer sex, access prevention and treatment, and experience health. Visual artmaking enables the artmaker to link head, heart, and hand and, in so doing, promote a “flow state.” According to Csikszentmihalyi (12), a flow state in the practice of artmaking is a sense of full immersion in an activity that the person enjoys. This encourages the artmaker to be more objective about their own experience as the image becomes the filter for that recalled memory (13, 14). Visual image making is not new in dialogues around health. Visual art-based methods offer advantages for young women to express the complexities of their S&RH experiences, promoting positive self-reflection and identity (14). “Body mapping,” a visual map created by tracing around the edge of the artist's physical body edge and rendering the lived experiences of the artist both within and outside that body space, has successfully been used as a youth sexual health intervention (15). The “body mapping” methodology employs a full-body image on one page, with all information delivered on the one body. Art-based research methods like these decrease perceived power imbalances between participants and researchers, enabling participants to voice their experiences and knowledge, lessening shame, and empowering the participants (13, 16, 17).

In this study, we explored a novel art-based methodology—called “Stories from the Edge”—to engage young women about how they navigated barriers to S&RH communication and how the process of artmaking assisted them to articulate their experiences. Coupled with established verbal methods like individual in-depth interviews (IDIs), visual art-based approaches could ease barriers to these difficult conversations.

## Methods

### Participants

In 2021, 12 young women were recruited through the Desmond Tutu Health Foundation Youth Centre (DTHF YC), all of whom had participated years earlier in a Wishing4Wellness (W4W) extramural programme exploring barriers to S&RH using visual art-based methods with the same art facilitator (FH; 2016–2017). Of these, seven were interested in this study and provided written informed consent to participate. The young women had limited prior experience with art and painting through formal schooling and brief exposure to painting in the afterschool Wishing4Wellness program in 2016–2017. The study was approved by the ethics committee of the University of Cape Town's Human Research Ethics Committee (HREC 368/2019).

### Conceptual framework

A socio-ecological conceptual model was used to map the trajectory of the study (18), which places the individual at the core of their worldly experiences and acknowledges the personal, familial, social, and institutional influences that shape their understandings of how they are able to express their needs and desires in seeking S&RH and treatment.

### Study design

This “Stories from the Edge” study consisted of five visual art-based sessions, each capturing the series of topics that may have influenced the S&RH experiences of young women (Table 1): (1) young women's sources of S&RH information pre- and post-puberty, (2) S&RH practices and promotors, (3) intimate relationships, (4) barriers to accessing S&RH treatment and wellness, and (5) S&RH service experiences and barriers. Between sessions 1 and 2, we included a session introducing painting and the concept of self-agency, which included colour mixing, creating textures, and a discussion of form, metaphor, and symbols. This session also introduced the concept of self-agency and how painting stories could contribute to communication of experiences and knowledge. At the end of the painting sessions (described below), IDIs were conducted with each young woman individually at the DTHF YC, in a private setting, which were audio recorded and transcribed for data analysis.

**Table 1:** Topics of the five “Stories from the Edge” sessions.

Session	Topics	Socio-ecological model (SEM) themes explored
1	Sources of sexual and reproductive health (S&RH) information pre- and post-puberty	Self and interpersonal
2	S&RH practices and promotors and intimate relationships	Self, interpersonal, community, and institutional relationships
3	Barriers to accessing S&RH care	Self, interpersonal, community, and institutional relationships
4	S&RH service experiences and barriers	Community and institutional
5	Individual in-depth interviews (IDIs)	Individual recall of the memories of experiences of S&RH journey as captured in paintings, recorded, and transcribed

### Individual in-depth interviews

A broad interview guide was used for the IDIs, with open-ended questions that focused mainly on each sphere of the socio-ecological conceptual model (family, friends and partners, community and culture, healthcare providers, and facilities) and the influences these had on individual S&RH experiences. Each young woman's paintings were displayed chronologically during the interview to prompt recall and how she had made meaning of her S&RH experiences during the painting process (8). The display of the young woman's paintings encouraged her to talk to what she saw in the images and consider how this related to her S&RH experiences. The paintings were photographed for digital recall. During the IDIs, the young women did not always explain their images but focused on what the images reminded them of their experiences. By providing a more objective view of their own experiences, each young woman was able to disentangle her feelings and emotions of those experiences and then gain a new perspective. All IDIs were conducted in English, although the young women were offered an isiXhosa translator if they preferred. IDIs were audio recorded and transcribed verbatim. Each young woman checked her own IDI transcript for accuracy. The interviewing researcher made field notes immediately after each interview and reflexively documented what she considered her position as an outsider, brought to the study.

### Description of the painting sessions

“Stories from the Edge” was developed to be participatory, to create a safe, non-judgemental delivery, with group support, to overcome language barriers and promote embodied recall, addressing each of the five topics described above. These sessions were intended to promote self-reflection, their self-esteem, and their creative thinking through the flow state experience that painting promotes (12). This offered the young women an opportunity for the painting process to generate new ideas about their experiences that they may not have reflected on previously. No previous painting skills were needed, and the materials offered were inexpensive poster paints, khokis, and pastels.

A large floor space was used for painting at the DTHF YC, with sufficient space for body-sized pieces of paper and social mingling. As with body mapping, participants traced the edges of their bodies onto large pieces of paper (sized 2 m × 1.5 m to A0). However, in “Stories from the Edge,” the body was not drawn in its entirety, but an edge was selected, and traced by another participant, to represent a chosen aspect of S&RH that the young woman wished to focus on. The body edge created a visual, personal, and physical edge on which to anchor her memories of her specific experiences. This body edge was achieved by lying down on a large piece of paper in a self-chosen position and has another participant trace around the young woman. The body edge was incorporated into the painting, often delineating what was sensed within the body space, and that which happened outside the physical body.

Each painting session represented a facet of the personal experiences of the young women, and new sheets of paper were used for each perspective of the emerging story, according to the session guides 1–5 (Table 1). Prior to each painting session, a focused introductory, participant-led discussion about the day's topic took place. These informal discussions were broadly focused on the topic of the day. Although these were recorded, they have not been transcribed and do not form part of the data set for this paper. The participants painted their stories, prompted by the discussion and broad open-ended questions. Each ensuing painting focused on another aspect of their S&RH experience (Table 1). To encourage more confidence in their art-based expression between sessions 1 and 2, the art-trained facilitator showed them various paintings by female artists. These paintings spoke of feelings within the body (not just literal translations of what happens to the body) and showed the young women that there was no “right” way of painting their feelings. Showing these paintings to the young women encouraged them to think about being in a body, having a body, and the emotional relationship with it and the world and opened up an alternative space for them, as evidenced in the ensuing paintings which used their own personal symbols and metaphors and were visually more abstract. An experienced female painter also visited (session 2) and discussed in a non-directed way how she came to make her own paintings. Having discussed various aspects of the paintings by the other artists, such as the visual vocabulary, use of symbols, metaphors, and the observed freedom to express feelings in many different ways, the young women felt encouraged to work more freely on their own works.

### Visual exploration

Although there was no prescriptive outcome intended from the painting sessions, each of paintings was viewed by an independent external art-trained facilitator (JT), who based her interpretation on the “process” rather than the “product” of each painting. Although we have not included analysis of the art works in this study, but rather an external exploration of the images, the process of visual analysis and its limitations are described by Ulman (19). The external viewer's notes on this exploration of the paintings related to the session topics and the anonymised information shared in the IDIs.

### Data analysis

The photographed artworks and IDI transcripts were used as the primary data set for analysis, which included a total of 24 paintings and seven IDIs. Immersive reading of textual data from the IDIs together with the artwork guides and external visual analysis gave an insight of the perspectives of the cohort that influenced their navigated S&RH knowledge and services.

## Results

Seven young women aged 21–25 years participated in “Stories from the Edge” study, painting their S&RH experiences. All young women were isiXhosa-speaking and completed their secondary school education in Masiphumelele, Cape Town. Four of these young women grew up rurally in the Eastern Cape, arriving in Cape Town for secondary (high) school. Four still lived in Masiphumelele. Four were currently completing tertiary education in Cape Town. Four reported being financially independent, and four reported being in stable relationships. Four of the young women had babies, with two having their babies living with them in Cape Town and two sending their babies to live with their family in rural Eastern Cape.

The results discuss the context and value of an art-based methodology and then key themes that influenced S&RH experiences: sexual partnerships, reproductive health experiences, and self-agency.

## Context about the art-based process

**Figure 1:**
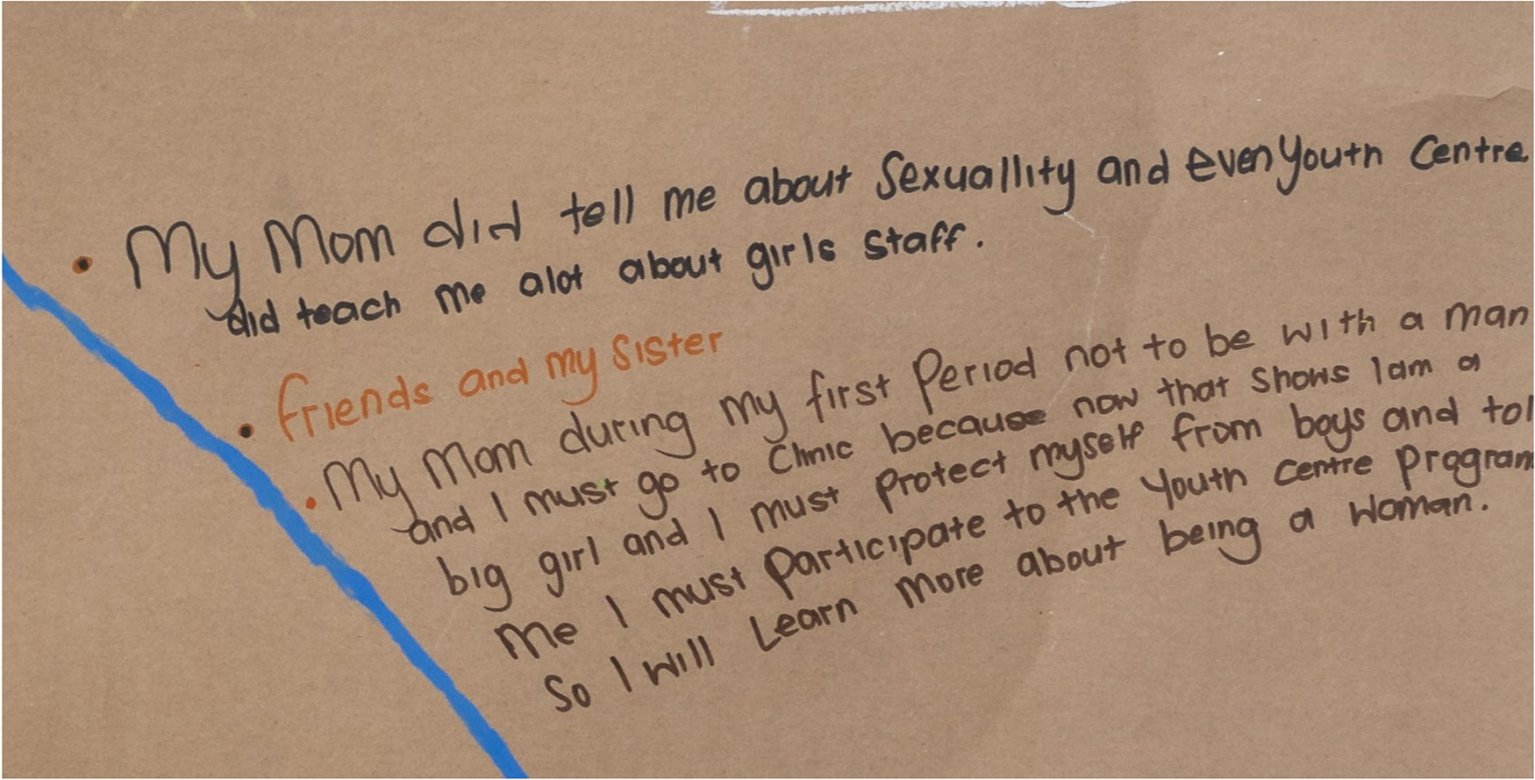
Language-led reflections about dominant sources of S&RH information, according to P09 (21 years old) in painting session 1, highlighting the role of her mother, friends, sister, and the adolescent healthcare facility in acquiring information.

**Figure 2:**
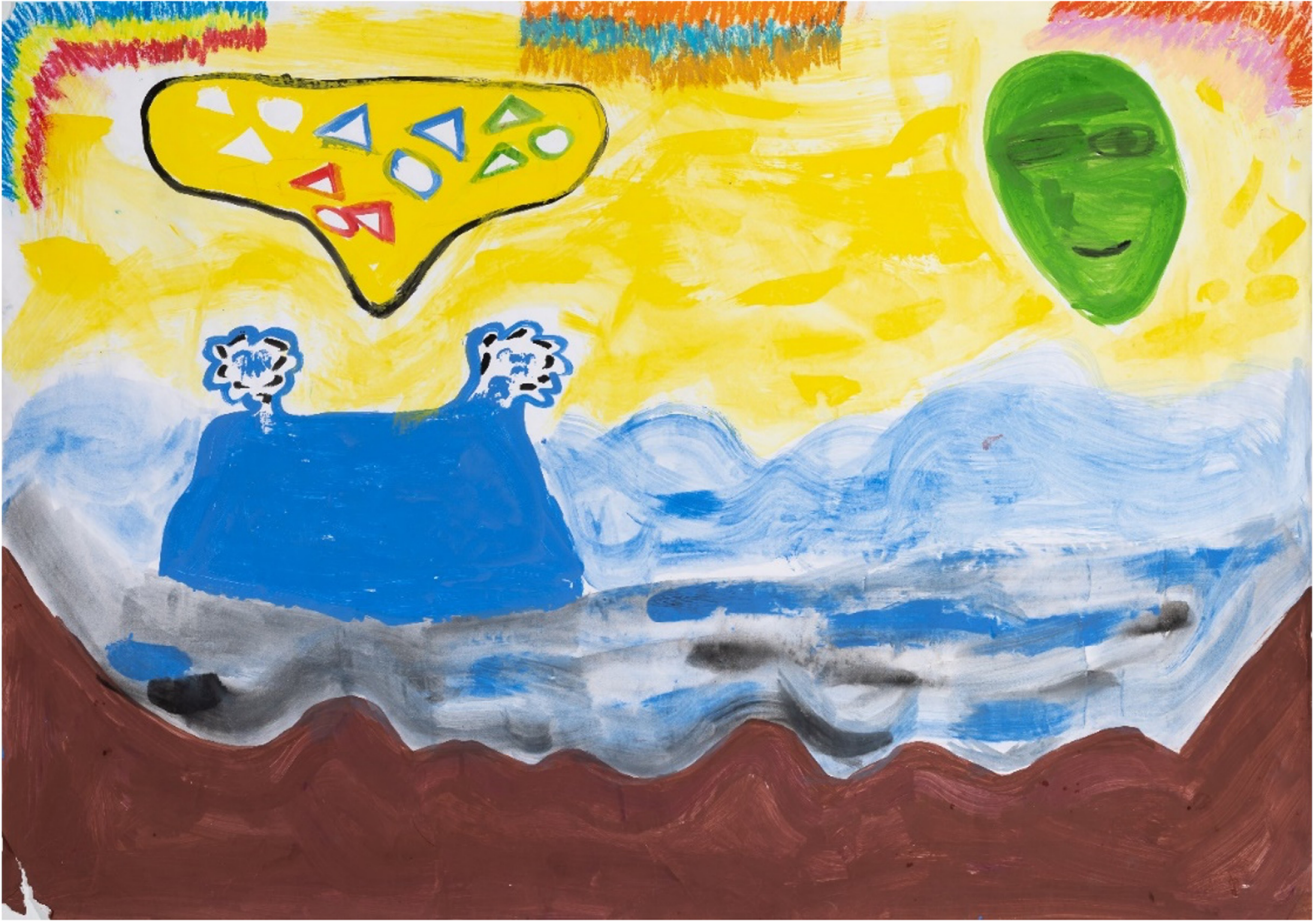
Reflections on S&RH health services experiences by P01 (25 years old), during painting session 5. Colour was used to represent depression (brown) with attending such a healthcare facility. She explained that the sun and clouds metaphorically capture relationship variabilities, with the sun suggesting that the positive experiences outshone the negative.

**Figure 3:**
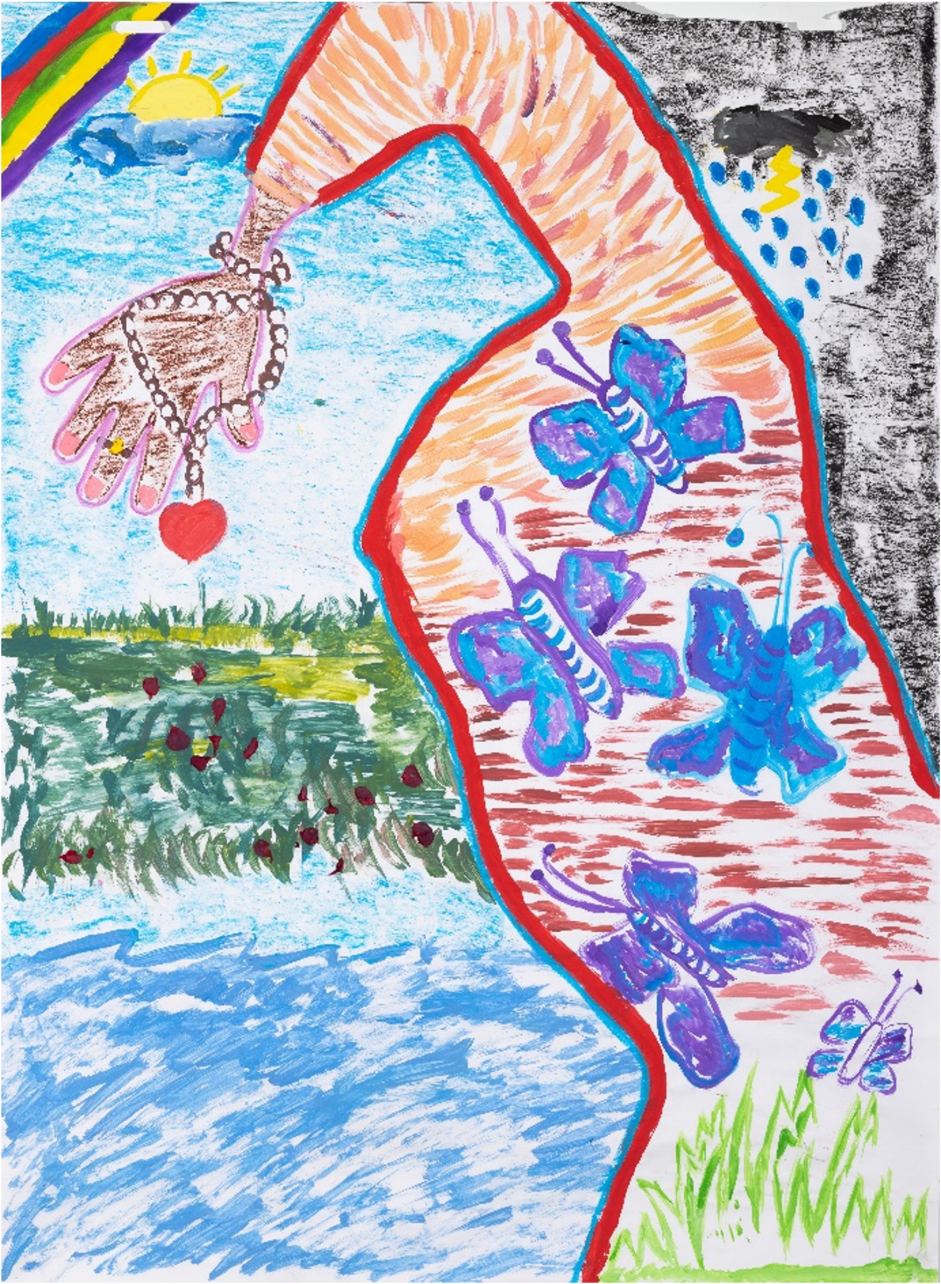
Reflecting on S&RH practices and promotors within intimate relationships, P03 (21 years old, painting session 5) illustrated the idea of being shackled to the idea of love, the little chain, and locket not only suggesting this, but it also suggests the jewellery that one gets from your lover.

**Figure 4:**
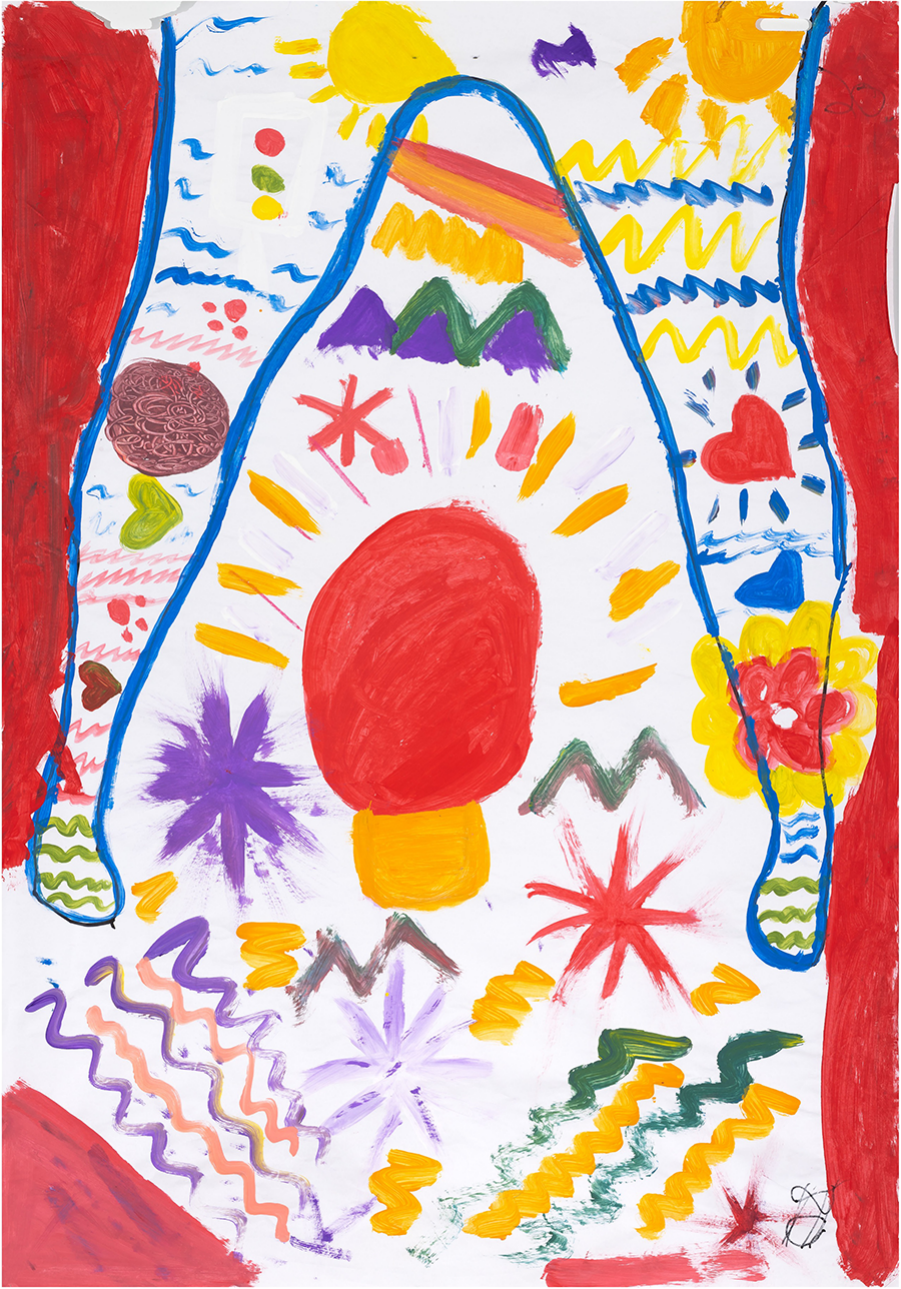
Considering S&RH practices and promotors within intimate relationships, P06 (23 years old, painting session 5) illustrated her painting that depicts a globe (the world) between her legs, as a symbol of the power of what she had to offer.

**Figure 5:**
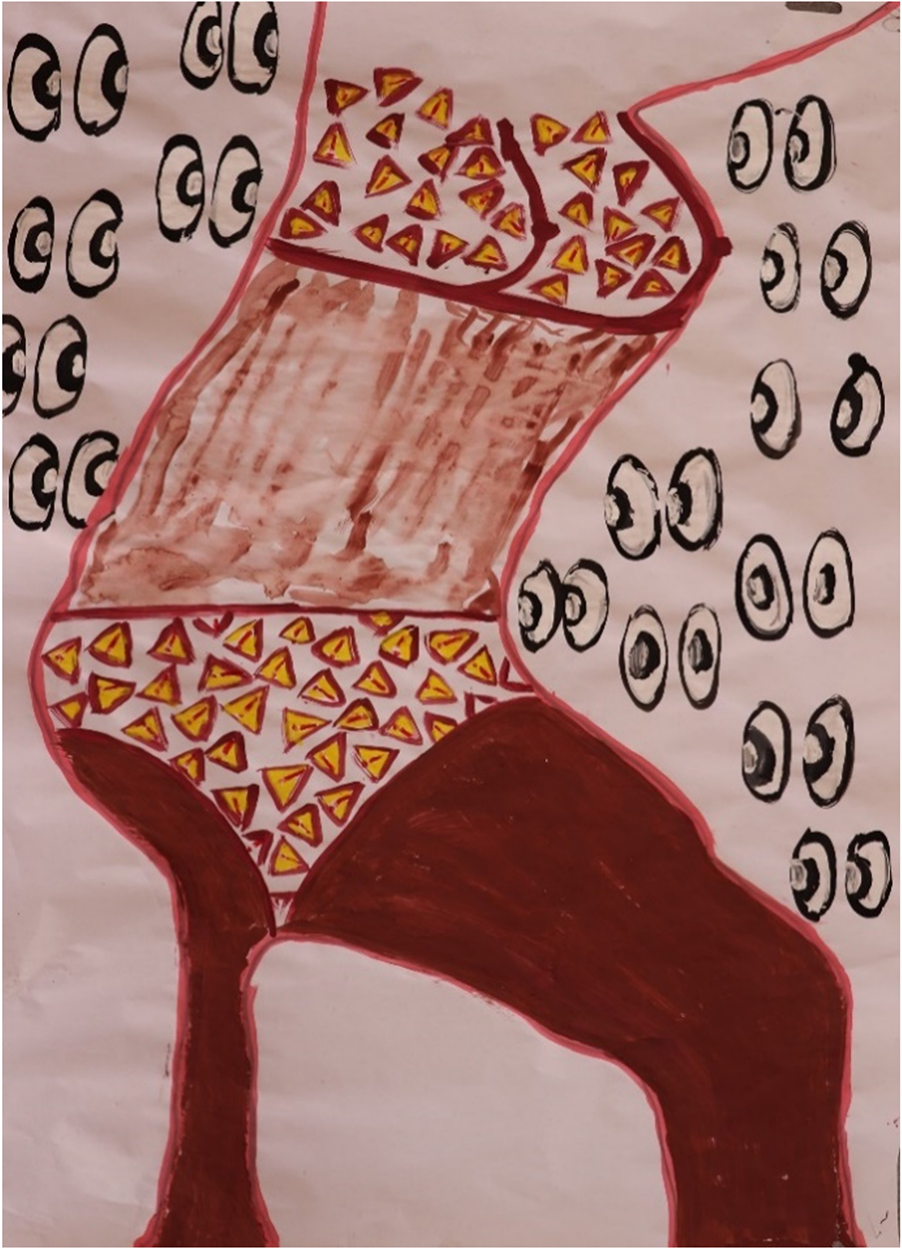
Exploring barriers to S&RH in painting by P03 (21 years old) in painting session 4. This young woman felt judged by the community for her unplanned pregnancy and a failed partnership. All-judging community eyes focused on her breasts (with aroused nipples longing for a good relationship) and genital area, carrying a stern warning: “we are always watching.”

**Figure 6:**
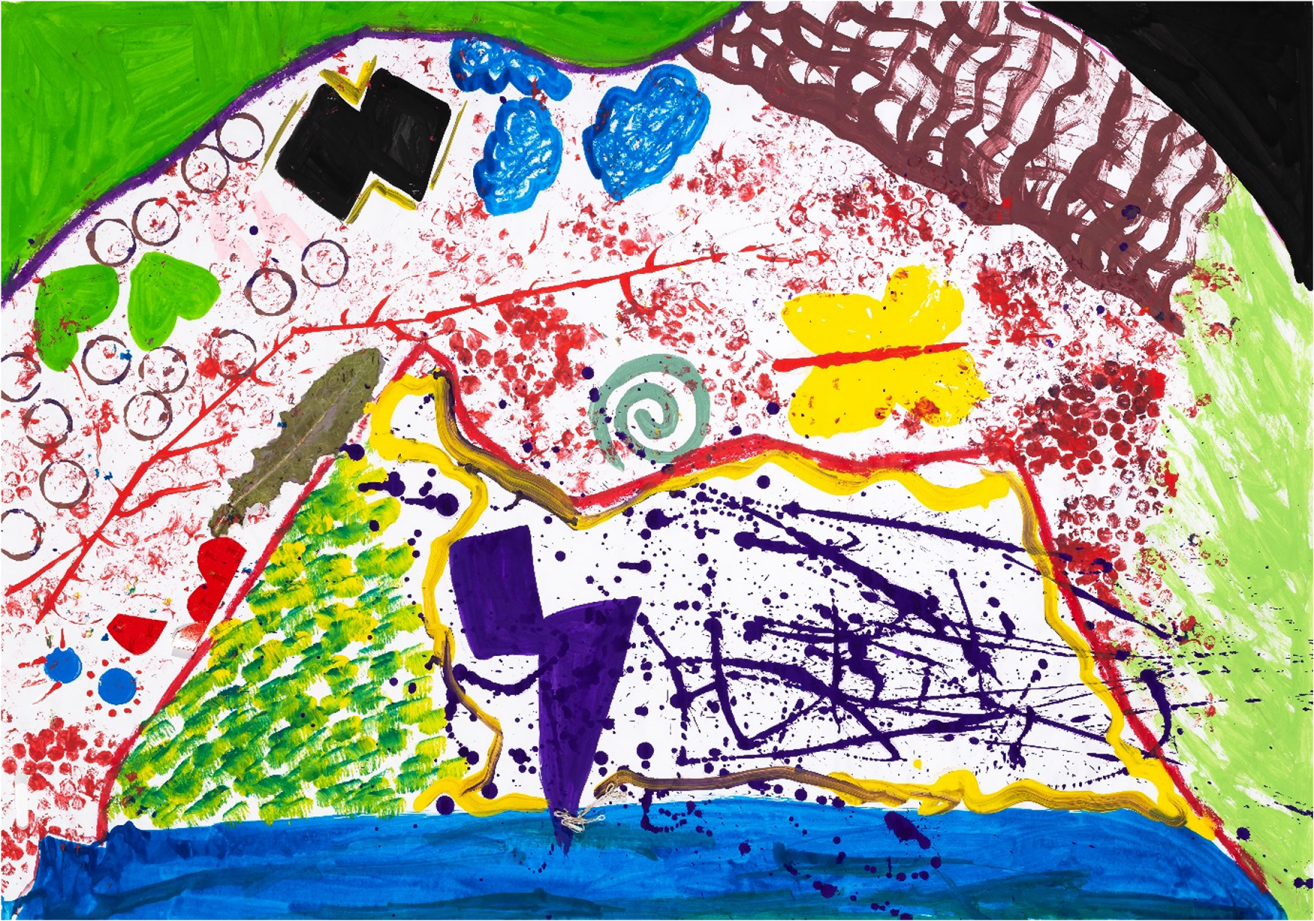
Art-based representation of a satisfying sexual relationship created by P09 (21 years old) in painting session 3, capturing the exuberance and confidence experiencing a good intimate relationship the body presence, is embedded in the body edge and shifts from foreground to background, shapes are strong and confident and mark-making free and experimental. Triangular shapes press in onto the body which holds its own with ease and grace.

**Figure 7:**
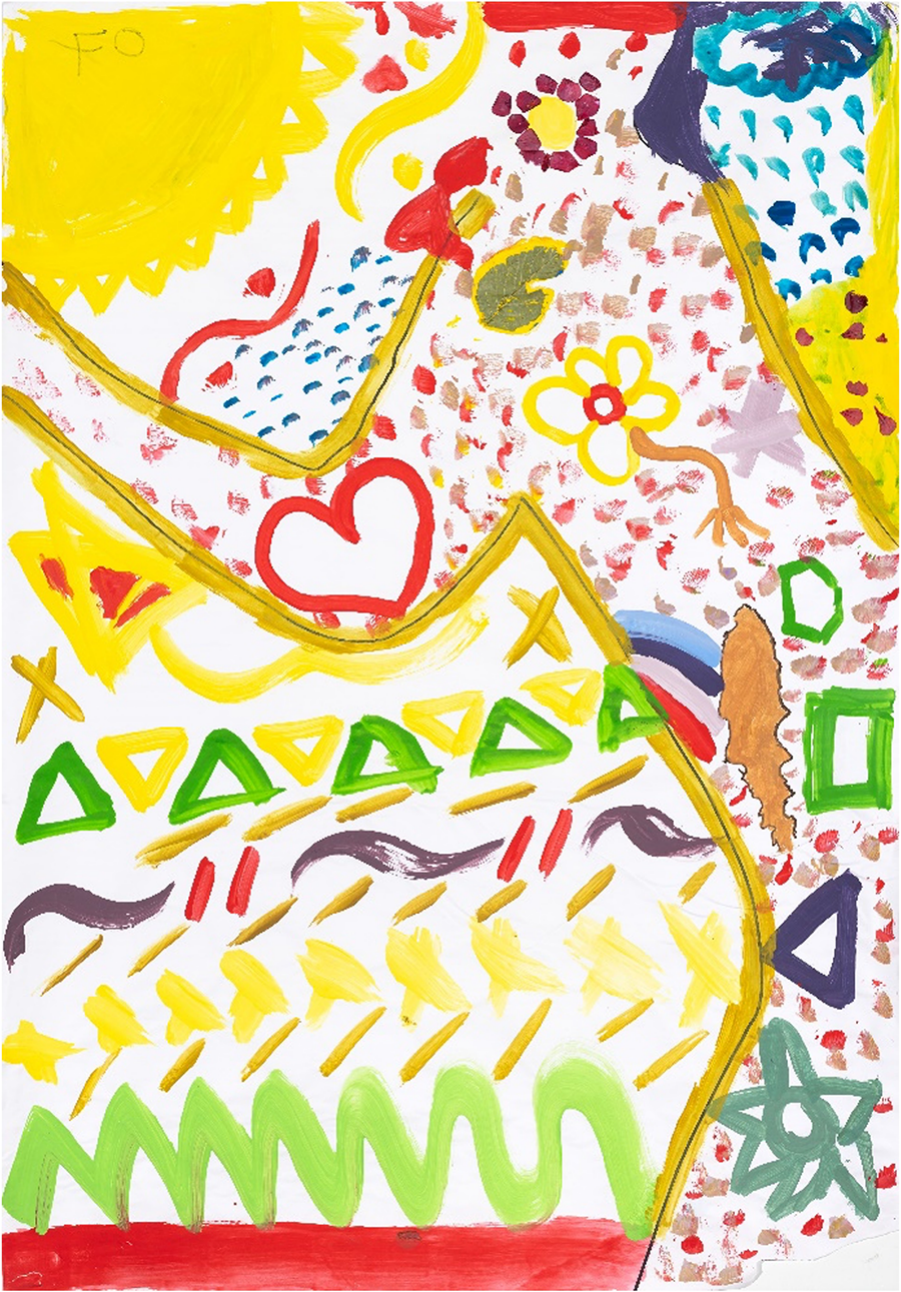
Reflections on intimate relationships, facilitators, and practices captured by P07 (23 years old) in painting session 3. Here, her body has rain clouds on the one side and the sun and patterns on the other side of a traced body edge. The decorative painting has a flow of marks and a limited use of colours. One shape is leaf-like, like the stem of the flower, the little hand-like root exploring downward, and what is below seems to be almost floating up to it.

**Figure 8:**
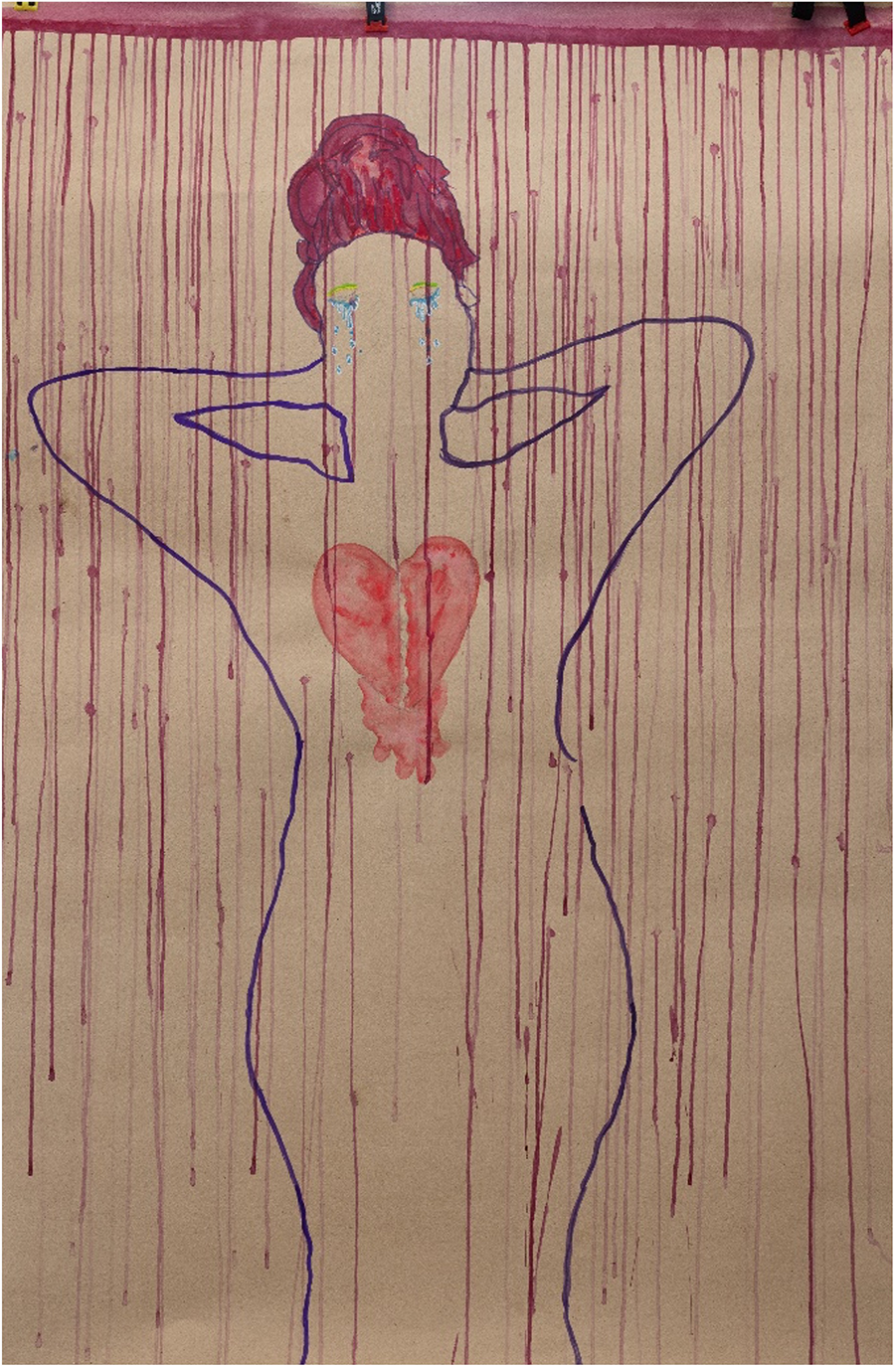
S&RH service experiences captured by P03 (21 years old) in painting session 5. The simple isolation of her body on the page, only an outline with carefully worked eyes, bold hair, and a watery soft heart quietly submitting itself to the pain while breaking and shrouded behind a curtain of red tears visually suggest that S&RH services do not offer much for a broken heart.

**Figure 9:**
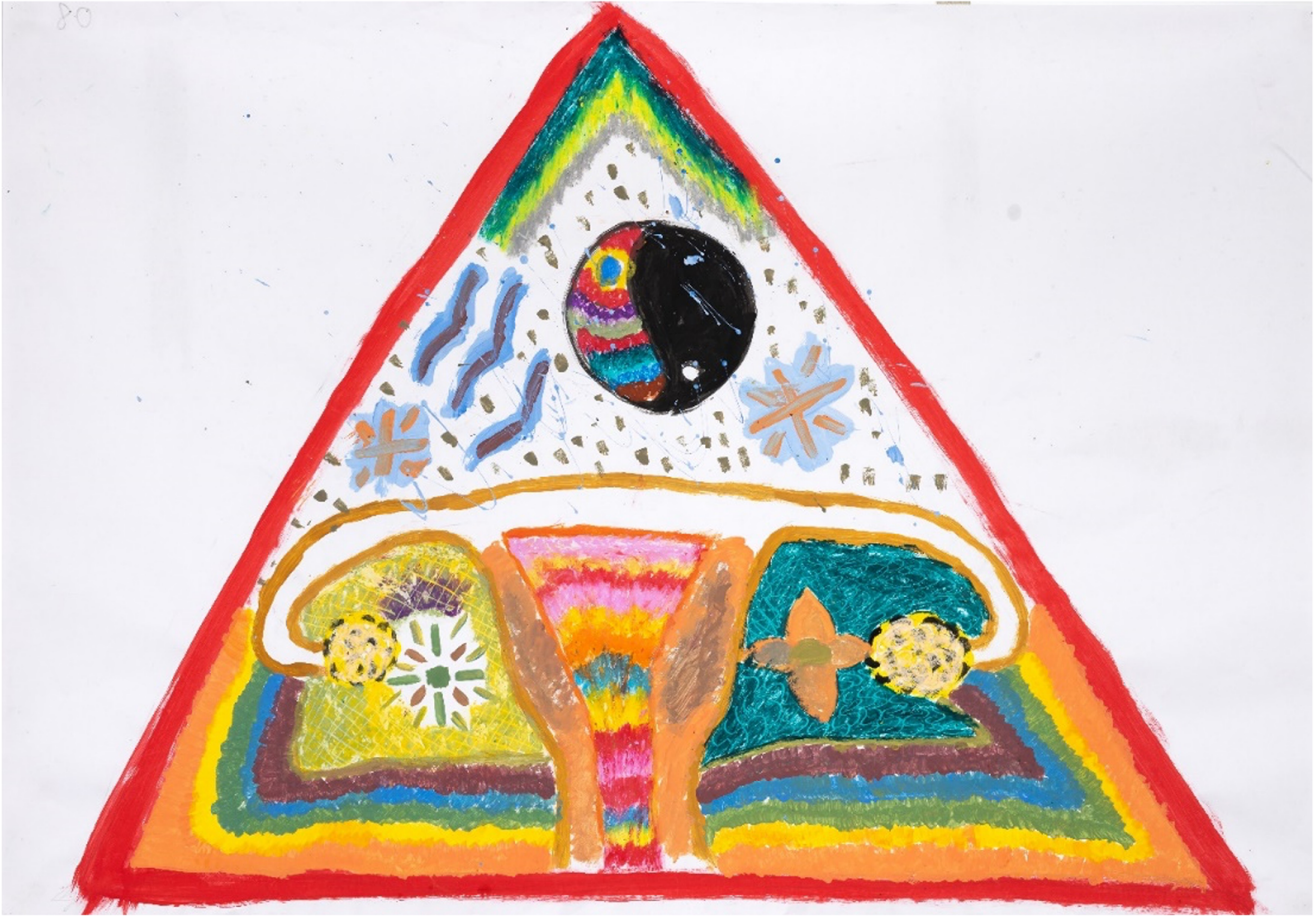
Reflections on intimate relationships as part of barriers to S&RH, captured by P08 (25 years old) in painting session 4, which captures her prioritising her own S&RH: it is her body, and no one has access to it without her permission. The triangle was intended as a warning sign, protecting her uterus and ovaries.

Stories from the Edge extended the body mapping method of using a full-body tracing to an edge of the body to ground the recalled experience in the body. At the beginning of the study, all of the young women expressed that they were not confident in their ability to make art but did enjoy painting. The full-body tracing in their first painting during session 1 (Figure 1) resulted in word-heavy verbal and visual generalisations. Thereafter the young women chose to use a body edge as a tool to visually demarcate and situate the feelings generated in response the focused S&RH topic of the session. This body edge was achieved by lying down on a large piece of paper in a self-chosen position and has another participant trace around the young woman. The body edge was incorporated into the painting, often delineating what was sensed within the body space, and that which happened outside the physical body. As their confidence in artmaking grew, some of the young women produced more abstract art-lead paintings (Figures 2–9) and lessened the use of the body edge. Some of the young women expressed that they found the process of painting their emotions difficult at times although they enjoyed that they could keep their emotions private by employing forms and symbols that they chose to use. To ease the process and encourage those who were finding it difficult, the facilitator used open questions during each session that prompted deeper reflection, intended to encourage a painted response of emotions into art. Informal chatting within the group while painting was a benefit, promoting sharing of experiences, S&RH knowledge, and friendships and social learning.

Over time, they appeared to enjoy expressing themselves freely, showing confidence in colour mixing, and creating more abstract forms that also allowed them to keep their experiences more private and personal. Importantly, as their confidence in the process developed, the obvious generalisations of word-dominant delivery of sensitive information diminished, and the more personal emotions of the experiences were grappled with and came to the fore in their paintings depicted in personal symbols and mark-making. The images they produced showed resilience and an ability to navigate their world, hoping for better communication about their S&RH needs and desires for more satisfying relationships (Figures 6, 7, and 9).

Figure 2 (P01, session 5) illustrates the power of artmaking as an enabler for difficult S&RH dialogues. Commenting on her painting, this young woman said that she represented her depression by the brownness in this image, although attending each artmaking study session (the routine, sense of sisterhood, and shared experiences) had alleviated her depression. She further disclosed that she felt that this was a safe place to relieve stressful emotions. The value of painting for P01 was difficult to reduce to words, as she explained about her feelings about her intimate relationship with her partner:

Interviewer: *…*“like the feelings, if you were going to write the words for those feelings?”

Participant*:* “They weren’t going to come out.”

Interviewer: “They weren’t?”

Participant: “They weren’t going to come out.”

Interviewer: “Yeah. And now that they are out, can you find the words for those feelings?”

Participant: “With, with words, that is too small.” (P01, 25 years old)

She continued:

“So, words like are really small for me, they are too small. And for words, I have to write each and everything and you could never be…write it to detail. But when you painting down… you get…like you have the image and all the feeling, you just be like, okay, this is what I’m going through, this is how I have been feeling …this color goes with my feeling.”

“without talking to someone I could really put down what I’m feeling and feel like I’ve…you know when you off load to someone” (Interviewer: yeah) “you turn to feel no load on you. But also, what I have noticed without you not talking to people, just telling yourself ‘I am chatting, I am actually chatting with this paper now that is in front of me,’ this paper, with these colours, with these brushes right in front of me, so it is the best.”

She said that she did not need a body edge to situate her feelings. Paint allowed her to express her feelings in a way that went beyond the limits of language. On the other hand, she also confessed that although she did have a mental image of what she wanted to capture in her painting, her painting skills did not do her imagination credit because for her hands:

“It was too hard.”

She still felt that she managed to express her feelings and acknowledged that she felt lighter afterward: her shoulders felt lighter, and the feelings were out of her body:

“I needed those colours to take it out of me.”

Another young woman commented, while insisting that she was not artistic, that:

“I feel like drawing is easy than writing when you express your feelings.” (P07, 23 years old)

Although this paper does not intend to provide an in-depth analysis of the IDI data collected, we have reflected on the manner in which the process of artmaking enabled difficult conversations about S&RH and recall of experiences by the participants with reference to the individual art works and the transcripts from each young woman's IDI, around three selected themes.

## Theme 1: relational dynamics influencing how young women gained S&RH knowledge and experiences

Close family, particularly mothers, grandmothers, and aunts, were focal sources of S&RH information during pre-adolescence for most young women, although only two of the young women had discussions with their caregivers (mothers, grandmothers, and aunts) about menstruation. Instead, there was reliance on clinic-sourced information about S&RH, as depicted in the word-dominant artwork by P09 telling of advice from her mother during her first menstrual period: that she “must not be with a man,” that she “must go to the clinic,” and that she “needs to protect [herself] from men.”

Early information about S&RH used fear mongering, primarily focusing on the prevention of pregnancies as one young woman relayed the advice from her grandmother, which came up in the IDIs although not evident in the painting generated about this topic:

“If you go with the guys and then you go sleeping with the guy, your cookie will be burnt…don’t go for guys…it's nice to have fun but don’t go….” (P01, 25 years old)

Many of the young women acknowledged in their IDIs that they accommodated their partners by not insisting on condom use, thereby putting themselves at risk to pregnancy and STIs to benefit from feeling “loved,” receiving material benefits such as gifts, enjoying the status of having a partner, and gaining possible protection from harm. This was reflected in Figure 3 (P03, session 3); the idea of being shackled to the idea of love, the little chain, and locket not only suggesting this but also suggesting the jewellery that one gets from your lover. In addition, this was captured by P06 in her painting (Figure 4), which the young woman explained while painting depicts a globe (the world) between her legs, as a symbol of the power of what she had to offer. Prompted by her painting, this same young woman commented that the responsibilities of condom use need to be shared:

“…then I was like: No man, where is the condom? And then he said: “Hayibo (meaning “oh no”) [P06] you see mos [filler word] there was no condom so why you asking now, after we done it?” So sometimes we, also girls, we have a lack of communication. It's not about boys who don’t want condom. We also don’t want condom… Maybe you [we] don’t speak. We also have to speak.” (P06, 23 years old)

Personal experiences of infidelity were a strong sub-theme that taught these young women to guard their hearts and bodies. As one young woman stated:

“Guys hurt you and then just leave.” (P03, 21 years old)

P03’s painting (Figure 3) from session 3 could be interpreted to reflect this lost love and betrayal quite literally: “A partial body edge was traced. The top right corner has a black background, a solid black cloud with lightning bolt, and raindrop tears in the storm. But there is a rainbow, sun, and clear sky on the opposite side that the body leans into, hoping for another relationship, though she now has a baby daughter and does not trust men. She feels bound to the possibility for a meaningful intimate partnership, as reflected in the butterflies in her body (for her a symbol of excitement, ‘butterflies in the stomach’), and new growth at the base of this artwork. But there is also the chain around her wrist, currently not binding her to another, but ready to submit to this perceived bondage of love. The natural stretch in this painting shows she is so part of her world, her universe internally and externally. Glued red petals, like teardrops, fall from the charmed heart. The deep internal space, and the spaces on both sides of the body reflect one another, creating a suspended visual tension, mirroring the tension of hope and wariness of positive intimate relationships.” External interpretation of meanings within another's paintings is clearly a subjective practice, particularly in this study which was intended to prioritise the process of artmaking rather than the product. However, from the artist’s perspective, the resilience of these young women, living in hope, is represented in paintings such as this.

Four of the seven young women had children of their own, for which they considered that they were judged by family and community.

Led by the painting she generated in session 4 (Figure 5), P03 revealed in her IDI that she felt judged by the community for her unplanned pregnancy and a failed partnership. She (P03) captured these feelings by depicting all-judging community eyes focused on her breasts and genital area. This painting could also be interpreted by the outside viewer as “carrying a stern warning: ‘we are always watching’.” The simplicity and directness of the message in this painting is strong and confident, where the external viewer interpreted that “one leg is giving the torso strength, the other less so.”

Despite the apparent obstacles that these young women faced around navigating their sexual debuts and sexual experiences, most women expressed a desire for a satisfying sexual relationship. One young woman commented:

“I think they [other young women] must put their selves first, love their selves, get more knowledge about life and their whole body, relationships, so that if they find their selves in a situation where they can't get out, they know, I did get this little bit of [information], from somewhere. So, I need to do this to get out.” (P09, 21 years old)

An example is also shown in her artwork (P09) which captures the exuberance and confidence of one young woman experiencing a good intimate relationship (Figure 6). This young woman (P09) said that she feels confident about caring for herself, and where her boundaries are, she can enjoy her (long-term) relationship. This painting could be interpreted by an external viewer as “the body presence being visually captured in the body edge and that the shift from foreground to background suggests a strong sense of self and how to protect herself, arching over what appears to be water in the foreground and an area of playfulness.” It also appears from this image that “She feels in control of her body,” which she verbally confirmed. From an artist's perspective, it was evident in Figure 6 that there are strong, confident shapes and mark-making, with free and experimental use of considered and controlled techniques. A further interpretation could be that “The top triangular shapes press onto the body which holds its own with ease and grace, suggesting her capacity to assert her desires and expectations for satisfying relationships.” In response to this painting during her IDI, the young women who produced this artwork (P09) stated:

“…now I’m like more ‘this is what I want from a relationship, like I want someone who's here to support me, love me… 50/50 of doing things, not like I'm expecting more from him; but here to love you and to support you through, or grow together.’ It has changed a lot… (from her younger self).” (P09, 21 years old)

Figure 7 metaphorically captured a young woman's comments about her feelings of intimate relationships, facilitators, and practices (P07 session 3). She outlined her body with rain clouds on the one side and the sun and patterns on the other side of a traced body edge. Her decorative painting has a flow of marks and a limited use of colours. There is a sense of her witnessing herself. From the perspective of an artist, one leaf-like shape in this figure is beautifully worked, like a piece of jewellery or a piece of flesh with a raw edge that has been touched, like the stem of the flower, the little hand-like root exploring downward, and what is below seems to be almost floating up to it. An interpretation of this painting could be that she “has been brave enough to explore in paint (leaf print and apparently random patterns), filling a space because she appears not to like it blank.” Reflecting on this painting during her IDI, she stated the following about her own S&RH experiences:

“…it is what it is.” (P07, 23 years old)

## Theme 2: reproductive health experiences at healthcare facilities

Fear of unintended pregnancy was the primary motivation for attending healthcare facilities, although paintings produced from the session on S&RH facilities (session 5) and the associated discussions during the IDIs highlighted the young women's feelings of shame, betrayal of confidentiality, and judgement by the facility staff for being sexually active. For example, the painting shown in Figure 8 references the experience of P03 when accessing S&RH services. In her IDI, reflecting on this painting, she commented:

“Yes, people tend to be harsh sometimes at [public] clinics but at the end of the day, you do get help.” (P03, 21 years old)

An outside interpretation of this particular painting may note that “the simple isolation of her body on the page, only an outline with carefully worked eyes, bold hair and a watery soft heart quietly submitting itself to the pain while breaking and shrouded behind a curtain of red tears, visually suggest that S&RH services do not offer much for a broken heart.” This participant’s words (from the IDI) and painted response regarding public S&RH services suggest that these clinics deliver on services but with little time to support her emotional wellbeing.

P01 in Figure 2, in the same session (session 5) appears to say little about S&RH healthcare facilities, which in itself could be considered a comment. This young woman used colour to represent her depression (brown) with attending such a healthcare facility. In her IDI, she further explained that the sun and clouds metaphorically capture relationship variabilities, with the sun suggesting that the positive experiences outshone the negative.

## Theme 3: self-agency and finding the words to communicate S&RH needs

When viewing their paintings, all the young women were pleased both with themselves and their artwork. For them, it mirrored how they had matured and how they had found the ability to express feelings and experiences which they had not been able to verbally express adequately. They said that they had learnt about themselves, witnessed their own growing, and felt more able to voice their own desires and needs because they understood themselves more fully. The young women reflected that they could witness their own evolving self-agency through their paintings, with one young woman commenting that painting allowed her to:

“Speak to the paper.” (P09, 21 years old)

From which, she elaborated:

“…just drawing it in whatever symbol you can, when it comes to that certain problem you have or [a] certain the answer you had to the certain question. So, drawing it meant you letting it go, but not necessarily letting the next person know what actually is going on with you.”

The process of artmaking created a safe space to explore their personal S&RH experiences in a visual way, as another young woman commented:

“Words are too small.” (P01, 25 years old)

This is indeed the reason to make art.

One young woman summarised this as:

“I feel like most important for me it has been growth and understanding, who I am and what I want in life. … who I am as a person, instead of who…what people think I am as a person.” (P08, 25 years old)

This young woman felt that this was accompanied by a lessening need for external validation, continuing:

“…understanding who I am and what I want in life.”

This young woman shows in her artwork (Figure 9 from session 4) that she is prioritising her own S&RH: it is her body, and no one has access to it without her permission:

“…the red on the outside was… don’t infiltrate if you’re not going to be serious about that.”

Casual relationships, without mutual care and S&RH responsibility, were not an option for her. The triangle was intended as a warning sign, protecting her uterus and ovaries. Interestingly, it is also the universal symbol of the female.

Many of the young women reported in their IDIs an increasing ability to voice their needs, desires, and opinions with their partners and more agency in their treatment-seeking behaviour. Their realisation that others may differently choose did appear to prompt increased tolerance for other's opinions and actions.

One young woman was able to verbalise how the study had benefitted her:

Interviewer: “…what have you learnt from us that you’re going to take forward in your life? If there's anything?”

Participant: “For, for this, I am taking…what is it that I am taking?”

Interviewer: “Hmm. What did you learn about yourself and what of that can you apply to your life now?”

Participant: “I’ve learnt to be reliable to myself, I should say to, to…I’ve learnt to be…I’ve learnt to be me, to talk, I’ve learnt to express things that I don’t want, or I don’t like. I’ve learnt to know that also other people they have their opinions and their own experience, they have their own way of solving stuff.” (P01, 25 years old)

## Discussion

**“**Stories from the Edge” explored the usefulness of creative visual art-based methods to encourage young women to recall and voice their experiences of S&RH, overcoming communication barriers of stigma and fear of judgement. These young women experienced the opportunity to create paintings in a safe space, being able to “speak to the paper” and keep their thoughts and feelings private through the use of their own personal symbols and abstraction. The verbalising of the process and the product (the paintings) was assisted by the informal discussions and chatter during the painting session and then the IDIs. The young women were encouraged and given basic tools to make artworks, after which they used this space to make images in their own time, without a sense of outcome and without the inhibition of being corrected. As a flat structure, the support was made available to encourage conversation through image making. From the artworks and accompanying interviews, three primary themes emerged, around sexual partnerships, reproductive health experiences, and the growing self-agency of the young women, that impacted the young women's sense of self.

Reflecting on the benefits of the “Stories from the Edge” process, the painting sessions promoted a safe, flexible, and creative space within which the young women could self-investigate their S&RH experiences and reflect on them, before verbalising their recall. Prompted by the visual cues from their “Stories from the Edge” paintings, the young women were empowered to mediate their verbal delivery in the IDIs, by reflecting on their emotions captured during the painting and organising their thoughts prior to telling their stories. In turn, the IDIs gave the young women the opportunity to verbalise their experiences. We found that “Stories from the Edge,” like other art-based methods, overcame language barriers and distanced themselves from recalling sensitive experiences (17). The “Stories from the Edge” process aided in repressing verbal overshadowing and enhanced experiential embodied recall (13, 20). During the process, positive self-reflection and expression contributed to their identity development and sense of self-agency. Within this ambit, young women were able to discretely express their personal S&RH experiences, without shame or judgement, extending their agency over information disclosure. During the “Stories from the Edge” process, the researchers were able to holistically examine the experiences of the young women. The young women maintained their roles as primary interpreters of their paintings by discussing the memories of their S&RH experiences that looking at the paintings prompted, thus reducing researcher bias. It was interesting to note the clear shift in the S&RH advice given to young women from adolescence to adulthood—from family to peer and to partner—and to observe how they depicted this shift in their paintings. They initially captured their memories of being advised by their primary caregivers (usually their grandmothers or mothers) to stay away from boys when they reached puberty in their paintings from session 1, and expanded on during their IDIs. In later sessions, they expressed in their figures and IDIs that they were able to choose which advice to take and that they were the carers of their own bodies, thus showing their developing reflexivity and increasing self-agency. Gaining insights from these young women about their lived S&RH experiences through the process of “Stories from the Edge,” artmaking could enable deeper insight of how adolescent S&RH experiences are shaped (10).

The young women yearned for the confidence and communication skills to have non-judgemental discussions with their mothers and partners. However, previous studies have suggested that mothers and grandmothers often do not have the S&RH language themselves to discuss options with the young women nor the relevant current information (5, 21). Zuma et al. (22) and Wang et al. (23) further reported that this limited S&RH information, vocabulary, and agency experienced by young women made them vulnerable to unsafe sex practices and the acquisition of STIs. Those who were guided to healthcare facilities were fortunate. As others have reported (3, 24), these young women valued adolescent-friendly sites, where staff were non-judgemental and maintained confidentiality.

The development of the paintings during the study captured the young women's emerging self-agency, which could also be interpreted as a benefit of the process. The language-dominant body mapping-like paintings of the first session transformed during the five sessions, where their paintings became more considered, confident, and controlled. This mirrored the developing self-agency and self-care that the young women expressed in their IDIs. The young women felt braver and more willing to prioritise themselves and express their own desires and needs for S&RH practices.

The “Stories from the Edge” body edge tracing was intended to situate the embodied experience and provide a repository, grounding the boundary between the young women's internal emotional and external physical experiences. As the young women's confidence in painting grew, they relied less on the body edge to situate their interior feelings and those that were from their experienced external physical world. The later paintings reflected more of their emotions and became more abstracted, not relying on a body edge to situate their story. Reviewing their paintings during the IDIs, the young women could identify their roles and S&RH choices and what shaped the navigation of the barriers to their S&RH. “Stories from the Edge” also allowed the young women to tell of enjoying their sexual partnerships, what they learnt from their partners, their emerging self-agency, and the complexities of their evolving S&RH experiences.

This study had limitations that should be acknowledged. This qualitative study including seven young women living in Cape Town provided deep and interesting insights into their experiences navigating S&RH and services and was not intended to provide generalisable evidence for the broader community. The process was intensive and required a significant time commitment on behalf of the young women and the facilitation team. However, it was because of the time and intensiveness that communication barriers around stigma and fear of judgement could be overcome. The young women could manage their artmaking of their S&RH experiences in a manner that was unrushed and afforded them privacy, by choosing personal symbols, metaphors, and abstraction. This mitigated the possibility of prompting distressing feelings and emotions. It is also likely that the positive responses to the method experienced by this cohort were biased by the fact that they all acknowledged enjoying art prior to enrolling. Other prospective participants may not, which could possibly lead to bewilderment or frustration with the process of translating their feelings onto paper, similar to difficulties some people would have in finding the words to communicate their feelings. Therefore, a creatively educated and skilled facilitator is essential for this process to be replicated, as are the carefully framed open questions that were used to prompt deeper reflection. It is important that discussions that happened during artmaking and IDIs were participatory, not led by the facilitator, with an exchange of ideas and skills, and where the “artwork” rather than the “participant” was discussed. The paintings were also independently viewed by a practicing artist, to add additional insights and interpretations to a study that is embedded in the art process. As such, these interpretations are inherently subjective and idiosyncratic as an outsider does not have a true insight into the intention of the artist (19). Furthermore, although the young women used their paintings as prompts when discussing their S&RH experiences, to recall what those experiences felt like, they did not talk about the final images they had painted. This was intentional as the method was about the process of artmaking rather than the product.

In conclusion, we describe here the process that “Stories from the Edge” followed as an art-based methodology, with the underlying principles, assumptions, and limitations, which we hope challenges the boundaries between the disciplines of art, public health, and science in enabling conversations about S&RH issues for young women. It was clear from the paintings and interviews with young women that to prioritise their own S&RH, young women have to navigate difficulties that challenge their self-agency. Using visual art-based methods such as “Stories from the Edge” may assist in overcoming some of the communication barriers of how young women relate their S&RH experiences and ease the sensitivity of these discussions. This could add a valuable insight to both the young women and researchers of their holistic S&RH life experiences and promote the self-knowledge of young women in a participatory and non-judgemental way. The collaborative creative group environment forged trust and friendships, which positively contributed to their developing communication skills. Developing confident communication skills, where young women feel comfortable asserting their S&RH needs and desires, may also improve the uptake of prevention and treatment options. It will be important in future studies to analyse the data collected through the IDIs against the SEM framework and to explore the themes that emerge.

## Data Availability

The raw data supporting the conclusions of this article will be made available by the authors, without undue reservation.
